# Genotype-Specific ECG-Based Risk Stratification Approaches in Patients With Long-QT Syndrome

**DOI:** 10.3389/fcvm.2022.916036

**Published:** 2022-07-14

**Authors:** Marina Rieder, Paul Kreifels, Judith Stuplich, David Ziupa, Helge Servatius, Luisa Nicolai, Alessandro Castiglione, Christiane Zweier, Babken Asatryan, Katja E. Odening

**Affiliations:** ^1^Translational Cardiology, Department of Cardiology, Inselspital, University Hospital Bern, University of Bern, Bern, Switzerland; ^2^Department of Cardiology and Angiology I, Faculty of Medicine, University Heart Center Freiburg-Bad Krozingen, University of Freiburg, Freiburg, Germany; ^3^Department of Human Genetics, Inselspital, Bern University Hospital, University of Bern, Bern, Switzerland; ^4^Department of Physiology, University of Bern, Bern, Switzerland

**Keywords:** long-QT syndrome, genetic arrhythmia disorders, risk stratification, QTc, electrocardiogram

## Abstract

**Background:**

Congenital long-QT syndrome (LQTS) is a major cause of sudden cardiac death (SCD) in young individuals, calling for sophisticated risk assessment. Risk stratification, however, is challenging as the individual arrhythmic risk varies pronouncedly, even in individuals carrying the same variant.

**Materials and Methods:**

In this study, we aimed to assess the association of different electrical parameters with the genotype and the symptoms in patients with LQTS. In addition to the heart-rate corrected QT interval (QTc), markers for regional electrical heterogeneity, such as QT dispersion (QT_max_-QT_min_ in all ECG leads) and delta T_peak/end_ (T_peak/end_ V5 – T_peak/end_ V2), were assessed in the 12-lead ECG at rest and during exercise testing.

**Results:**

QTc at rest was significantly longer in symptomatic than asymptomatic patients with LQT2 (493.4 ms ± 46.5 ms vs. 419.5 ms ± 28.6 ms, *p* = 0.004), but surprisingly not associated with symptoms in LQT1. In contrast, post-exercise QTc (minute 4 of recovery) was significantly longer in symptomatic than asymptomatic patients with LQT1 (486.5 ms ± 7.0 ms vs. 463.3 ms ± 16.3 ms, *p* = 0.04), while no such difference was observed in patients with LQT2. Enhanced delta T_peak/end_ and QT dispersion were only associated with symptoms in LQT1 (delta T_peak/end_ 19.0 ms ± 18.1 ms vs. −4.0 ms ± 4.4 ms, *p* = 0.02; QT-dispersion: 54.3 ms ± 10.2 ms vs. 31.4 ms ± 10.4 ms, *p* = 0.01), but not in LQT2. Delta T_peak/end_ was particularly discriminative after exercise, where all symptomatic patients with LQT1 had positive and all asymptomatic LQT1 patients had negative values (11.8 ± 7.9 ms vs. −7.5 ± 1.7 ms, *p* = 0.003).

**Conclusion:**

Different electrical parameters can distinguish between symptomatic and asymptomatic patients in different genetic forms of LQTS. While the classical “QTc at rest” was only associated with symptoms in LQT2, post-exercise QTc helped distinguish between symptomatic and asymptomatic patients with LQT1. Enhanced regional electrical heterogeneity was only associated with symptoms in LQT1, but not in LQT2. Our findings indicate that genotype-specific risk stratification approaches based on electrical parameters could help to optimize risk assessment in LQTS.

## Introduction

Congenital long-QT syndrome (LQTS) is an inherited arrhythmia disorder with an estimated prevalence of 1:2000 (1). Pathogenic variants in genes encoding cardiac ion channel subunits or channel-interacting proteins lead to delayed and dispersed cardiac repolarization, the underlying mechanism for the prolonged QT interval observed in the surface electrocardiogram (ECG) of patients with LQTS (2). The altered repolarization predisposes to ventricular arrhythmias, potentially leading to syncope and sudden cardiac death (SCD) (3). Despite its rarity, LQTS is a major cause of SCD in apparently healthy young individuals, indicating the importance of reliable risk stratification to reduce the SCD burden (4).

Pathogenic variants in one of the three major genes associated with LQTS, namely, *KCNQ1*, *KCNH2*, and *SCN5A*, account for most of the mutation-positive cases (5, 6): 40–55% of patients have LQTS type 1 (LQT1) due to loss-of-function variants in *KCNQ1*, which encodes the α-subunit of the K^+^-channel K_*V*_7.1, generating the repolarizing outward K^+^-current I_*Ks*_ (3). I_*Ks*_ is activated by adrenergic stimuli and physiologically leads to a shortening of the QT interval when heart rate increases (7). A diminished function of I_*Ks*_ in LQT1 is associated with particularly prolonged repolarization, thus pronouncing arrhythmic risk in the context of high sympathetic tone (3, 7). Around 30–45% of mutation-positive LQTS cases are caused by loss-of-function variants in *KCNH2*, which encodes the α-subunit of the HERG K^+^-channel generating the rapid delayed rectifier I_*Kr*_ current (LQTS type 2, LQT2) (3). Arrhythmic events in patients with LQT2 are frequently associated with startle and sudden sympathetic surge (8, 9). Around 5–10% of all mutation-positive patients with LQTS have LQTS type 3 (LQT3), caused by gain-of-function variants in *SCN5A* (3, 10). *SCN5A* encodes the α-subunit of the cardiac sodium channel that conducts the depolarizing sodium inward current (I_*Na*_), and its gain-of-function increases late I_*Na*_ and thereby prolongs cardiac repolarization (11). In contrast to LQT1 and LQT2, arrhythmic events in LQT3 frequently occur at rest or sleep (7, 12). Despite the knowledge regarding genotype-specific pro-arrhythmic triggers, these are not incorporated into the individual patients’ risk stratification schemes.

Genetic testing can identify the molecular substrate and thus reveal genotype-specific pro-arrhythmic conditions (7, 13). The individual patients’ arrhythmic risk, however, varies markedly, even within families carrying the same variant (14, 15). This makes the individual patient’s risk stratification a challenging task, especially the indication for antiarrhythmic treatment or the implantation of a cardioverter defibrillator (ICD). Heart-corrected QT interval (QTc) in combination with clinical data (age, gender, history of arrhythmic syncope, or ventricular arrhythmias) and information on the underlying genotype are currently factors considered for risk stratification; however, predicting the individuals’ arrhythmic risk, especially in so far asymptomatic patients remains a difficult task (16–18). Incorrect risk assessment may lead to either overtreatment of low-risk patients with anti-arrhythmic medication or ICD or to an underestimation of risk, resulting in the occurrence of ventricular arrhythmia with possibly devastating consequences. This highlights the need for novel parameters that outperform the currently used QTc in predicting the individual’s risk.

There is emerging evidence that the arrhythmic risk in LQTS is not only due to delayed repolarization depicted by a prolonged QTc interval (17). Instead, the spatial and temporal alteration of repolarization is significantly involved in arrhythmia formation (19). Novel ECG parameters that reflect the temporal and spatial heterogeneity of repolarization, such as the short-time variability of QT (STVQT), T_peak/end_, or the inter-lead variability of QT duration (QT dispersion), have been described to be associated with arrhythmic risk in small patient cohorts with LQTS (20–22). However, little is known about genotype differences regarding these parameters and their usefulness in risk prediction.

In this study, we, therefore aimed (a) to assess electrical parameters not only at rest but also in the context of pro-arrhythmic conditions such as exercise and (b) to add parameters of spatial (and temporal) heterogeneity of repolarization for genotype-specific risk stratification compared with the established parameter QTc in patients with LQTS.

## Materials and Methods

Between 2020 and 2022, patients with genetically confirmed LQTS presenting at the Genetic Arrhythmia Clinic at the University Hospital of Bern (Inselspital) were included in this analysis. The study protocol was approved by the Cantonal Ethics Committee of Bern (ID 2016-01602 and ID 2020-00316). All participants had provided written informed consent prior to the analysis of their data.

### Study Population

Patients with pathogenic or likely pathogenic variants identified in one of the three major LQTS genes (*KCNQ1*, *KCNH2*, and *SCN5A*) were included in the analysis. These patients presented at the Genetic Arrhythmia Clinic at Inselspital Bern for genetic counseling, testing, or routine clinical follow-up examinations. Variant interpretation followed the principles and recommendations outlined in the 2015 statement of the American College of Medical Genetics and Genomics/Association of Molecular Pathology (23). Twelve-lead ECG, exercise ECG and Holter ECG monitoring for at least 24 h were or had been performed as part of clinical routine examinations based on the decision of the treating physician. All 12-lead ECGs were recorded at 25 mm/s with standard lead positions. Exercise testing was carried out according to the standard or modified Bruce protocol, depending on the patient‘s physical capacity (24). The patients’ genotypes including the underlying causal variant, the clinical history, and the medications were recorded, especially the use of beta-blockers or any other antiarrhythmic treatment.

Patients with ventricular tachycardia, presyncope/syncope, or aborted cardiac arrest were assigned to the “symptomatic” group. All other participants were allocated to the “asymptomatic” control group.

### Assessment of Electrical Parameters

We analyzed 12-lead ECGs, exercise ECGs and Holter ECGs to study the association of different electrical parameters with the genotype and the clinical phenotype.

In standard 12-lead ECG recordings (obtained at rest in a supine position), we determined the QT interval and the RR interval with the corresponding heart rate in lead II. QT intervals were measured from the onset of the QRS complex to the end of the T wave, which was defined as the intersection between the isoelectric baseline and the tangent line of the sloping T wave (25). When the T-wave had a biphasic or notched configuration, the tangent was set behind the last slope. QT intervals were corrected for heart rate using Bazett’s formula to obtain the heart rate corrected QT interval (QTc) (26). Despite its limitations, we used Bazett’s formula (27–29) since it remains the preferable formula for heart-rate correction of the QT interval in LQTS and is used in most LQTS studies (30). In the case of pacemaker stimulation, the Bogossian formula was additionally applied (31). In addition to the standard electrical parameter QTc, we assessed markers for regional electrical heterogeneity such as QT dispersion, which represents the dispersion of ventricular repolarization (22). We determined the inter-lead variation of QT as the difference between the longest and shortest QT values measured in each of the 12 ECG leads (QT_max_–QT_min_) (22). Furthermore, we analyzed the interval between peak and end of the T wave (T_peak/end_) in leads V2 and V5. T_peak/end_ reflects transmural dispersion of repolarization and was determined as the interval between the peak of the T-wave to the end determined by the tangent method (25). We then calculated the novel parameter delta T_peak/end_ (T_peak/end_ V5 – T_peak/end_ V2) to depict the regional and transmural electrical heterogeneity in the region of the anterior wall, an area that was described to be particularly affected by repolarization abnormalities in LQTS (32). All parameters were determined in three consecutive beats, and the mean was used for further analyses.

Similarly, all measurements described above (QTc, QT dispersion, T_peak_/_end_, and delta T_peak/end_) were also performed in the stress ECG before start of the exercise (standing position) and at minute 4 of the post-exercise recovery phase. Again, three consecutive beats were analyzed and averaged for each parameter.

Additionally, the short time variability of QT (STVQT), which reflects the temporal dispersion of repolarization (20), was determined at 9 am (± 1 h) in the Holter ECG and during exercise ECG before start of exercise and at minute 4 of the recovery phase. STVQT was analyzed, and Poincaré plots were obtained by plotting the QT interval against the previous interval for 31 beats as previously described (33, 34).

All measurements were performed manually by a single observer (M.R.) to avoid inter-operator variabilities.

### Validation Cohort

Patients with genetically confirmed LQT1 or LQT2 presenting at the University Hospital of Freiburg, Germany, between 2015 and 2017 were included in the validation cohort. The protocol for the validation cohort was approved by the institutional ethics committee from the University Hospital of Freiburg (Germany) (ID 479/14). All patients had received Holter ECG monitoring for at least 24 h. We calculated STVQT according to the measurements in the identification cohort from the University Hospital Bern (Inselspital). Patients were classified as symptomatic or asymptomatic according to the cohort from the University Hospital Bern.

### Data Analysis

Statistical analyses were performed using GraphPad Prism 9 (GraphPad Software, San Diego, United States). Significant outliers were excluded using Gubb’s test. Continuous variables were tested for normal distribution by using the Shapiro–Wilk test. As QTc, QT dispersion, T_peak/end_, delta T_peak/end_ and STVQT followed a Gaussian distribution, data are presented as mean ± SD. A comparison of the two cohorts was performed using Student’s *t*-test. For the comparison of the different LQTS genotypes, ANOVA with Tukey’s multiple comparisons test was performed.

Receiver operating characteristic (ROC) was performed to evaluate with which sensitivity and specificity it was possible to distinguish between symptomatic and asymptomatic patients with different electrical parameters.

A two-tailed *p*-value less than 0.05 was considered statistically significant.

## Results

A total of 28 patients (53.5% female, mean age 37 years) with genetically confirmed LQTS, including 11 with LQT1 (39.3%), 13 with LQT2 (46.4%), and 4 with LQT3 (14.3%), were included in the study between 2020 and 2022.

The patients with LQTS carried the following variants in *KCNQ1* (NM_000218.3): p.(Arg366Gln) (2 patients), p.(Arg231Cys) (6 patients), p.(Arg249Gly), and p.(Asp242Asn) (2 patients); the following variants in *KCNH2* (NM_000238.3): p.(Asp509Asn), p.(Arg1512Trp), p.(Arg1014*), p.(Arg863*) (3 patients), p.(Ala561Val) (2 patients), p.(Arg531Trp) (3 patients), p.(Glu698*), and p.(Arg176Trp); and the following variants in *SCN5A* (NM_198056.2): p.(Arg1512Trp), p.(Gln1507_Pro1509del) (2 patients), and p.(Asn1774His).

In the LQT1 group, five patients (45.5%) had experienced presyncope, syncope, ventricular tachycardia, or symptomatic early-onset atrial fibrillation at an age of 16 and were therefore classified as symptomatic. Six patients (54.5%) had not presented any of these arrhythmic symptoms in the previous years and were therefore classified as asymptomatic. In the LQT2 group, five patients (38.5%) had a history of syncope or torsade-de-pointes tachycardia, while eight (61.5%) patients were classified as asymptomatic. Of the four patients with LQT3, one had previously suffered from torsade-de-pointes tachycardia and had received an ICD for secondary prevention. One had a pacemaker due to chronotropic incompetence. The other two were asymptomatic, however, one had received an ICD due to various cases of SCD in the family (Table 1).

**TABLE 1:** Patient characteristics.

LQT1		Symptomatic (*n* = 5)	Asymptomatic (*n* = 6)
	Age [years]	28.0 ± 16.5	45.7 ± 13.7
	Male	3 (60%)	4 (67%)
	Beta-blocker	3 (60%)	1 (17%)
	ICD	0 (0%)	0 (0%)
LQT2		**Symptomatic (*n* = 5)**	**Asymptomatic (*n* = 8)**
	Age [years]	39.4 ± 3.1	29.5 ± 19.8
	Male	0 (0%)	5 (63%)
	Beta-blocker	5 (100%)	6 (75%)
	ICD	5 (100%)	0 (0%)
LQT3		**Symptomatic (*n* = 2)**	**Asymptomatic (*n* = 2)**
	Age [years]	46.0 ± 21.1	53.0 ± 24.0
	Male	1 (50%)	0 (0%)
	Beta-blocker	2 (100%)	0 (0%)
	ICD/pacemaker	2 (100%)	1 (50%)

*Age is presented as mean ± standard deviation. The other parameters are presented as number of patients (with percentage based on the number of patients). Beta-blocker depicts the intake of beta-blockers at the time-point of the analyzed 12-lead-ECG. In the symptomatic LQT1 cohort, either propranolol, nadolol, or metoprolol were used. The asymptomatic LQT1 patient received metoprolol. In the symptomatic LQT2 cohort, either metoprolol, or bisoprolol were used. Patients in the asymptomatic LQT2 group received either propranolol, metoprolol, or atenolol. The symptomatic patients with LQT3 were prescribed atenolol and metoprolol. The two symptomatic patients with LQT1 without beta-blocker therapy at the time point of the 12-lead ECG have started beta-blocker treatment in the meantime.*

### Genotype-Differences

#### 12-Lead Electrocardiogram at Rest

**FIGURE 1:**
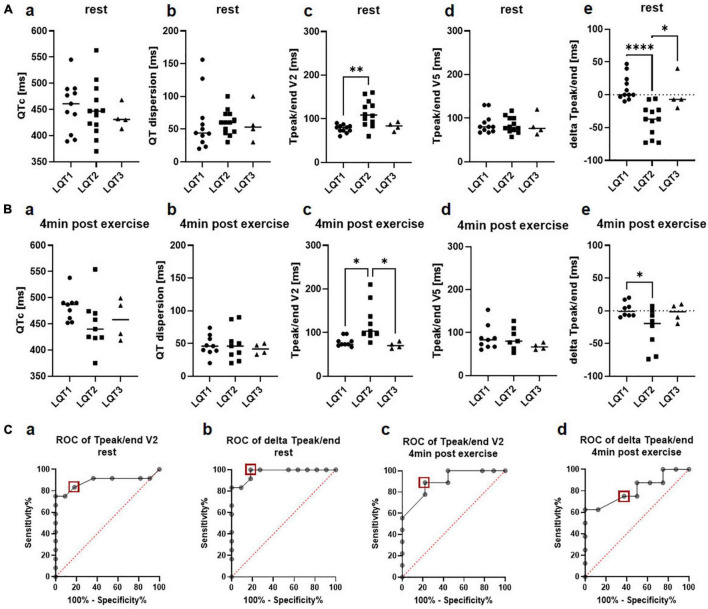
Genotype differences in electrical parameters. **(A)** 12-lead ECG at rest. **a** QTc at rest did not differ been the genotypes **b** QT dispersion was comparable between LQT1, LQT2, and LQT3 **c** T_peak/end_ in lead V2 was significantly longer in LQT2 compared to LQT1. **d** T_peak/end_ in lead V5 did not differ between genotypes **e** delta T_peak/end_ (T_peak/end_ V5 – T_peak/end_ V2) was significantly lower in patients with LQT2 compared to LQT1 and LQT3. LQT1: *n* = 11, LQT2: *n* = 13, LQT3: *n* = 4. *p < 0.05, ^**^p < 0.01, ^****^p < 0.0001 **(B)** Exercise-ECG 4 min after exercise. **a** QTc 4 min after exercise did not differ been the genotypes **b** QT dispersion post-exercise was comparable between LQT1, LQT2 and LQT3 **c** T_peak/end_ in lead V2 was significantly longer in LQT2 compared to LQT1 and LQT3. **d** T_peak/end_ in lead V5 did not differ between genotypes **e** delta T_peak/end_ (T_peak/end_ V5 – T_peak/end_ V2) was significantly lower in patients with LQT2 compared to LQT1. LQT1: *n* = 9, LQT2: *n* = 9, LQT3: *n* = 4. *p < 0.05. **(C) a** In ROC analysis, T_peak/end_ at rest in lead V2 had a sensitivity of 83% and a specificity of 82% to discriminate between LQT1 and LQT2 at a cut-off of 85ms (red square). **b** In ROC analysis, delta T_peak/end_ at rest had a sensitivity of 100% and a specificity of 82% at a cut-off of −4.5ms (red square) for discrimination between LQT1 and LQT2. **c** T_peak/end_ in lead V2 4 min post-exercise was identified as a poor marker with a sensitivity of 89% and a specificity of 78% to discriminate between LQT1 and LQT2 at a cut-off of 86.5ms (red square). **d** Delta T_peak/end_ 4 min post-exercise was a poor marker as well with a sensitivity of 75% and a specificity of 63% to discriminate between LQT1 and LQT2 at a cut-off of −6.5ms (red square).

In the 12-lead ECG at rest (lying position), QTc did not differ significantly between the LQTS genotypes (LQT1: 455.5 ms ± 48.2 ms, LQT2: 447.9 ms ± 51.0 ms, LQT3 435.8 ms ± 23.1 ms; Figure 1Aa). QT dispersion was also comparable in LQT1 (59.7 ms ± 43.2 ms), LQT2 (60.0 ms ± 18.7 ms), and LQT3 (59.0 ms ± 29.5 ms) (Figure 1Ab).

T_peak/end_ was significantly prolonged in the patients with LQT2 compared to the LQT1 cohort in lead V2 (LQT1 77.18 ms ± 8.9 ms, LQT2 112.8 ms ± 30.7 ms, LQT3 82.5 ms ± 9.9 ms, *p* = 0.002 for LQT1 vs. LQT2; Figure 1Ac), but no differences were observed in lead V5 (LQT1 87.6ms ± 22.9 ms, LQT2 84.3 ms ± 18.1 ms, LQT3 84.0 ms ± 25.0 ms, Figure 1Ad). ROC analysis revealed that T_peak/end_ in lead V2 only had a sensitivity of 83% and a specificity of 82% to discriminate between LQT1 and LQT2 genotypes at a cut-off of 85 ms (AUC 0.89, CI 0.7 to 1.0; Figure 1Ca).

In contrast, the novel marker delta T_peak/end_ was significantly higher in LQT1 and patients with LQT3 compared to the LQT2 group (LQT1 10.5 ± 19.2, LQT2 −39.3 ms ± 24.1 ms, LQT3 1.5 ms ± 26.4 ms, *p* < 0.0001 for LQT1 vs. LQT2 and *p* = 0.01 for LQT2 vs. LQT3, Figure 1Ae). In ROC analysis, delta T_peak/end_ performed better than T_peak/end_ alone with a sensitivity of 100% and a specificity of 82% at a cut-off of −4.5 ms for discrimination between LQT1 and LQT2 (AUC 0.97, CI 0.9 to 1.0; Figure 1Cb).

#### Exercise-Electrocardiogram

Comparable with the 12-lead ECG at rest, QTc did not differ significantly between the LQTS genotypes after exercise testing (minute 4 of the recovery period: LQT1 481.9 ms ± 26.4 ms, LQT2 449.2 ms ± 49.5 ms, LQT3 458.3 ms ± 39.7 ms; Figure 1Ba), even though it was numerically higher in LQT1 than in LQT2 or LQT3. QT dispersion was also comparable in LQT1 (47.0 ms ± 15.9 ms), LQT2 (48.7 ms ± 25.2 ms), and LQT3 (41.5 ms ± 8.3 ms) at minute 4 of the recovery phase (Figure 1Bb). In line with the 12-lead ECG at rest, T_peak/end_ in lead V2 was longer in LQT2 compared to LQT1 or LQT3 (LQT1 78.9 ms ± 11.1 ms, LQT2 123.8 ms ± 44.4 ms, LQT3 70.8 ms ± 7.4 ms, *p* = 0.01 for LQT1 vs. LQT2 and *p* = 0.02 for LQT2 vs. LQT3; Figure 1Bc). However, T_peak/end_ in lead V2 4 min post-exercise only had a sensitivity of 89% and a specificity of 78% to discriminate between LQT1 and LQT2 at a cut-off of 86.5ms (AUC 0.9, CI 0.8 to 1.0; Figure 1Cc).

T_peak/end_ in lead V5 again showed no difference between the genotypes (LQT1 88.2 ms ± 29.6 ms, LQT2 86.7 ms ± 26.2 ms, LQT3 67.5 ms ± 7.6 ms; Figure 1Bd). Delta T_peak/end_ was significantly reduced in LQT1 compared to LQT2 (LQT1 2.1 ± 11.6, LQT2 −28.4 ± 31.1, LQT3 −3.3 ± 14.2, *p* = 0.03 for LQT1 vs. LQT2, Figure 1Be). However, it was not a good parameter to discriminate between LQT1 and LQT2 with a sensitivity of only 75% and a specificity of 63% at a cut-off of −6.5ms (AUC 0.8, CI 0.6 to 1.0; Figure 1Cd).

Short time variability of QT as a marker of temporal electrical heterogeneity did not differ between the three genotypes, neither before start of the exercise (LQT1 8.9ms ± 4.3ms, LQT2 9.8ms ± 3.6ms, LQT3 8.3ms ± 4.0ms) nor at minute 4 of the recovery phase (LQT1 9.2ms ± 4.3ms, LQT2: 10.1ms ± 3.3ms, LQT3: 7.7ms ± 2.0ms), nor during Holter-ECG recordings (LQT1: 6.2ms ± 1.3ms, LQT2: 7.8ms ± 3.2ms, LQT3: 6.5ms ± 1.2ms).

### Genotype-Dependent Differences Between Symptomatic and Asymptomatic Patients

#### 12-Lead Electrocardiogram at Rest

**FIGURE 2:**
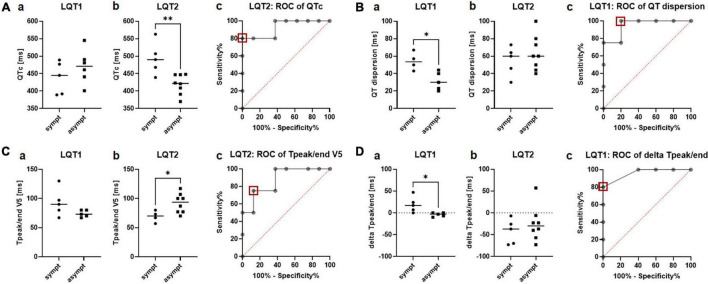
Electrical parameters in the 12-lead ECG at rest in symptomatic vs. asymptomatic LQT1 and LQT2 patients. **(A)** QTc at rest. **a** QTc did not differ between symptomatic (*n* = 5) and asymptomatic (*n* = 6) patients with LQT1. **b** QTc was significantly prolonged in the symptomatic vs. asymptomatic patients with LQT2. **c** ROC analysis of QTc [symptomatic (*n* = 5) vs. asymptomatic (*n* = 8)] in the LQT2 group. A cut-off of 458ms (red square) identified symptomatic patients with a sensitivity of 80% and a specificity of 100%. ^**^p < 0.01. **(B)** QT dispersion. **a** Symptomatic patients with LQT1 (*n* = 4) presented a significantly increased QT dispersion compared to the asymptomatic group (*n* = 5). **b** QT dispersion was comparable between symptomatic (*n* = 5) and asymptomatic patients with LQT2 (*n* = 8) **c** ROC analysis identified symptomatic patients with LQT1 with a cut-off of 41.5ms (red square) with a sensitivity of 100% and a specificity of 80%. *p < 0.05. **(C)** T_peak/end_ in lead V5. **a** T_peak/end_ did not differ between symptomatic (*n* = 5) and asymptomatic patients with LQT1 (*n* = 5). **b** T_peak/end_ was prolonged in asymptomatic patients with LQT2 (*n* = 8) compared to the symptomatic cohort (*n* = 4) **c** ROC analysis of T_peak/end_ in LQT2 revealed a cut-off of less than 75ms (red square) to distinguish asymptomatic and symptomatic patients with LQT2 with a sensitivity of 75% and a specificity of 87.5%. *p < 0.05. **(D)** Delta T_peak/end_ (T_peak/end_ V5 – T_peak/end_ V2). **a** Delta T_peak/end_ was higher in symptomatic (*n* = 5) than asymptomatic patients with LQT1 (*n* = 5). **b** No difference in delta T_peak/end_ could be observed between symptomatic (*n* = 6) and asymptomatic patients with LQT2 (*n* = 8). **c** ROC analysis revealed a cut-off of 3.5 ms of delta T_peak/end_ to detect symptomatic patients with LQT1 with a sensitivity of 80% and a specificity of 100%. *p < 0.05.

In the 12-lead ECG at rest, QTc was significantly prolonged in symptomatic compared to asymptomatic patients with LQT2 (493.4ms ± 46.5ms vs. 419.5ms ± 28.6ms, *p* = 0.004; Figure 2Ab), while not being associated with the clinical phenotype in LQT1 (438.4ms ± 46.6ms vs. 469.8ms ± 48.6ms; Figure 2Aa). ROC analysis revealed that a QTc of 458ms identified symptomatic patients with LQT2 in our cohort with a sensitivity of 80% and a specificity of 100% (AUC 0.93, CI 0.8 to 1.0; Figure 2Ac).

The marker for regional electrical heterogeneity, QT dispersion, was only associated with the arrhythmic phenotype in LQT1, where symptomatic patients had a significantly increased QT dispersion compared to the asymptomatic cohort (54.3ms ± 10.2ms vs. 31.4ms ± 10.4ms, *p* = 0.01; Figure 2Ba). ROC analysis showed that QT dispersion identified symptomatic patients with a cut-off of 41.5ms with a sensitivity of 100% and a specificity of 80% (AUC 0.95, CI 0.8 to 1.0; Figure 2Bc). In LQT2, QT dispersion was comparable between the symptomatic and asymptomatic groups (54.6ms ± 16.9ms vs. 63.4ms ± 20.1ms; Figure 2Bb).

In LQT1, no difference in T_peak/end_ could be observed between symptomatic and asymptomatic patients, both in lead V2 (73.8ms ± 8.9ms vs. 80.0ms ± 8.7ms) and V5 (92.8ms ± 23.7ms vs. 74.0ms ± 5.9ms; Figure 2Ca). In LQT2, T_peak/end_ was reduced in symptomatic patients in lead V5 (69.3ms ± 9.7ms vs. 91.9ms ± 16.6ms, *p* = 0.03; Figure 2Cb) but not in lead V2 (104.3ms ± 18.0ms vs. 117.1ms ± 35.7ms). ROC analysis revealed that T_peak/end_ V5 was not an ideal parameter to distinguish between symptomatic and asymptomatic patients. A cut-off of less than 75ms identified asymptomatic patients with a sensitivity of only 75% and a specificity of 87.5% (AUC 0.88, CI 0.7 to 1.0; Figure 2Cc).

In contrast, the novel marker delta T_peak/e*nd*_ (T_peak/end_ V5 – T_peak/end_ V2) was particularly distinct in symptomatic vs. asymptomatic patients with LQT1, with much higher values in symptomatic patients (19.0ms ± 18.1ms vs. −4.0ms ± 4.4ms, *p* = 0.02; Figure 2Da). ROC analysis revealed a cut-off of 3.5ms of delta T_peak/end_ to detect symptomatic patients with LQT1 with a sensitivity of 80% and a specificity of 100% (AUC 0.96, CI 0.8 to 1.0, Figure 2Dc). In contrast to LQT1, no difference in delta T_peak/end_ could be observed between symptomatic and asymptomatic patients with LQT2 (−42.6ms ± 28.5ms vs. −25.3ms ± 39.3ms; Figure 2Db).

#### Exercise Electrocardiogram

**FIGURE 3:**
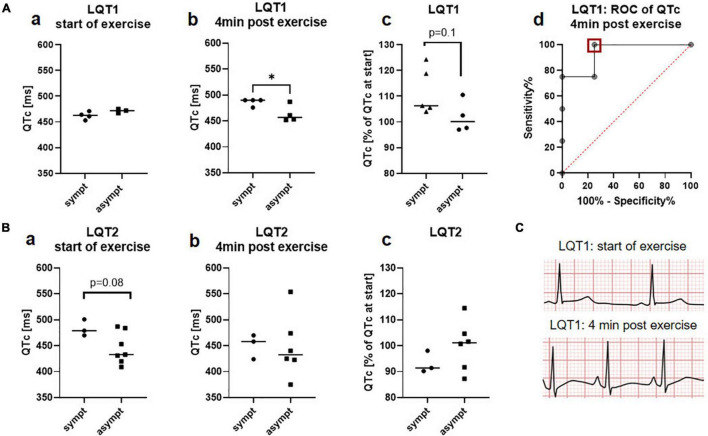
exercise-induced alterations of QTc depending on the clinical phenotype in LQT1 and LQT2. **(A)** LQT1 **a** QTc at rest did not differ between symptomatic (*n* = 4) and asymptomatic patients (*n* = 3) at start of exercise **b** 4 min after exercise, QTc was significantly prolonged in symptomatic patients with LQT1 (*n* = 4) compared to the asymptomatic cohort (*n* = 4) **c** QTc prolongation tended to be pronounced in the symptomatic (*n* = 5) compared to the asymptomatic patients (*n* = 4). **d** ROC analysis revealed a QTc cut-off of 488.5 ms to detect symptomatic patients with LQT1 with a sensitivity of 100% and a specificity of 75%. *p < 0.05. **(B)** LQT2 **a** QTc at rest (standing position) tended to be prolonged in the symptomatic (*n* = 3) compared to the asymptomatic patients at start of exercise (*n* = 7) **b** no differences in QTc were observed 4min after exercise in the symptomatic (*n* = 3) compared to the asymptomatic cohort (*n* = 6) **c** QTc prolongation did not differ between the symptomatic (*n* = 3) and asymptomatic patients **(C)** representative ECG (lead II) of a symptomatic LQT1 patient at start of exercise and at minute 4 of the recovery period with markedly exercise-induced QTc prolongation.

In contrast to the 12-lead ECG at rest, QTc tended to be prolonged in symptomatic patients with LQT2 compared to the asymptomatic cohort in the standing position (483.3ms ± 16.0ms vs. 445.3ms ± 30.6ms, *p* = 0.08; Figure 3Ba). In LQT1, no such differences could be observed prior to the beginning of exercise, consistent with the findings from the 12-lead ECG at rest (462.3ms ± 7.5ms vs. 471.3ms ± 4.0ms, Figure 3Aa). In contrast, 4 min after exercise, QTc-prolongation was not only observed in LQT1, but it tended to be nominally even more pronounced in symptomatic than in asymptomatic patients with LQT1 (111.8% ± 9.1% vs. 101.9% ± 6.2%, *p* = 0.1; Figure 3Ac), leading to a significantly longer post-exercise QTc in the symptomatic than asymptomatic cohort with LQT1 (486.5ms ± 7.0ms vs. 463.3ms ± 16.3ms, *p* = 0.04; Figure 3Ab), while no such differences were observed in the cohort with LQT2 (93.3% ± 4.3% vs. 100.1% ± 9.6%; Figure 3Bc; QTc post-exercise 450.7ms ± 23.9ms vs. 448.5ms ± 60.8ms; Figure 3Bb). According to ROC-analysis, a QTc of 488.5ms at 4 min post-exercise could discriminate between symptomatic and asymptomatic patients with LQT1 with a sensitivity of 100% and a specificity of 75% (AUC 0.9, CI 0.8 to 1.0; Figure 3Ad). A representative ECG of a symptomatic LQT1 patient at start of exercise and 4 min after exercise is depicted in Figure 3C.

**FIGURE 4:**
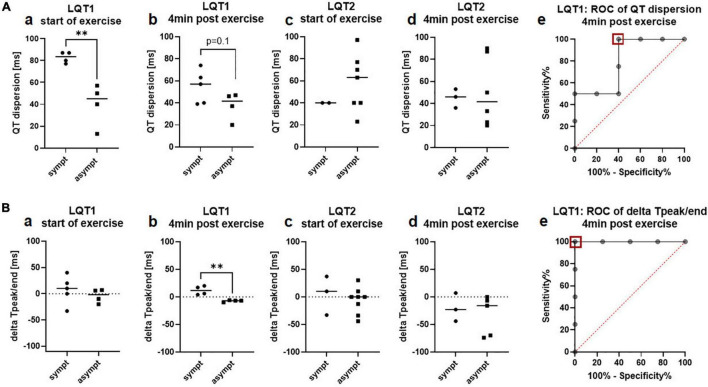
QT-dispersion and delta T_peak/end_ in the exercise-ECG depending on the arrhythmic phenotype in LQT1 and LQT2. **(A)** QT-dispersion. **a** QT-dispersion was significantly prolonged in symptomatic patients with LQT1 (*n* = 4) compared to the asymptomatic group (*n* = 4) at start of exercise. **b** At minute 4 of the recovery phase, QT dispersion still tended to be prolonged in symptomatic (*n* = 5) compared to asymptomatic (*n* = 4) patients with LQT1. **c** QT dispersion was comparable between symptomatic (*n* = 2) and asymptomatic (*n* = 7) patients with LQT2 at start of exercise. **d** QT dispersion did not differ between symptomatic (*n* = 3) and asymptomatic (*n* = 6) patients with LQT2 at minute 4 of the recovery period. **e** ROC-analysis: A cut-off of less than 52ms (red square) identified asymptomatic patients with LQT1 with a sensitivity of 100% and a specificity of 60%. ^**^p < 0.01 **(B)** delta T_peak/end_. **a** delta T_peak/end_ was comparable between symptomatic (*n* = 4) and asymptomatic (*n* = 4) patients with LQT1 at start of exercise. **b** 4 min after exercise, all symptomatic patients with LQT1 (*n* = 4) presented positive values, and all asymptomatic (*n* = 4) negative values **c** Delta T_peak/end_ did not differ between symptomatic (*n* = 3) and asymptomatic (*n* = 8) patients with LQT2 at start of exercise. **d** No difference in delta T_peak/end_ between symptomatic (*n* = 3) and asymptomatic (*n* = 5) patients with LQT2 was observed at minute 4 of the recovery period. **e** ROC-analysis: A cut-off of −1ms of delta T_peak/end_ 4 min after exercise (red square) can discriminate between symptomatic and asymptomatic patients with LQT1 with a sensitivity and a specificity of 100%. ^**^p < 0.01.

As observed in the 12-lead ECG at rest, QT dispersion was more pronounced in symptomatic than asymptomatic patients with LQT1, both at rest (82.8ms ± 5.1ms vs. 40.0ms ± 19.3ms, *p* = 0.005; Figure 4Aa) and nominally 4 min after exercise (54.6ms ± 15.1ms vs. 37.5ms ± 12.5ms, *p* = 0.1; Figure 4Ab). However, ROC analysis revealed that exercise-induced QT dispersion was not an ideal parameter to discriminate between symptomatic and asymptomatic patients with LQT1, as a cut-off of less than 52ms had a sensitivity of 100% for the identification of asymptomatic individuals, but only a specificity of 60% (AUC 0.8, CI 0.5 to 1.0; Figure 4Ae). In LQT2, there was no association with the clinical phenotype, neither at the start the of exercise (40.0ms ± 0.0ms vs. 58.6ms ± 25.6ms; Figure 4Ac) nor at minute 4 of the recovery period (45.0ms ± 8.5ms vs. 50.5ms ± 31.3ms; Figure 4Ad).

T_peak/end_ after exercise was not associated with the clinical presentation in both genotypes, neither in lead V2 nor V5 (LQT1: V2 78.6ms ± 10.9ms vs. 79.3ms ± 13.0ms; V5 101.4ms ± 34.0ms vs. 71.8ms ± 11.5ms; LQT2: V2 106.7ms ± 11.9ms vs. 132.3ms ± 53.3ms; V5 86.7ms ± 37.5ms vs. 110.0ms ± 55.0ms).

In contrast, delta T_peak/end_ was particularly discriminative after exercise, where all symptomatic patients with LQT1 had positive values and all asymptomatic patients with LQT1 had negative values (start of exercise: delta T_peak/end_ 7.4ms ± 27.0ms vs. −4.3ms ± 13.1ms; Figure 4Ba; 4 min after exercise 11.8ms ± 8.0ms vs. −7.5ms ± 1.7ms, *p* = 0.003; Figure 4Bb). ROC analysis revealed that exercise-induced delta T_peak/end_ was an optimal parameter for the discrimination of symptomatic and asymptomatic patients with LQT1. A cut-off of −1ms had an ideal sensitivity and specificity of 100% (AUC 1.0, CI 1.0 to 1.0; Figure 4Be). In LQT2, there was no association with the clinical phenotype, neither at start (4.7ms ± 35.3ms vs. −6.4ms ± 23.7ms; Figure 4Bc) nor at minute 4 after exercise (−20.0ms ± 25.6ms vs. −33.4ms ± 35.7ms; Figure 4Bd).

STVQT as a measure of temporal electrical heterogeneity was comparable between symptomatic and asymptomatic LQT1 and LQT2 patients, both before start of exercise (LQT1 11.0ms ± 4.8ms vs. 6.2ms ± 1.1ms; LQT2 9.7ms ± 2.7ms vs. 10.0ms ± 4.4ms) and at minute 4 of the recovery phase (LQT1 10.9ms ± 2.8ms vs. 7.0ms ± 5.2ms; LQT2 10.0ms ± 5.4ms vs. 10.9ms ± 0.3ms).

#### Holter-Electrocardiogram

**FIGURE 5:**
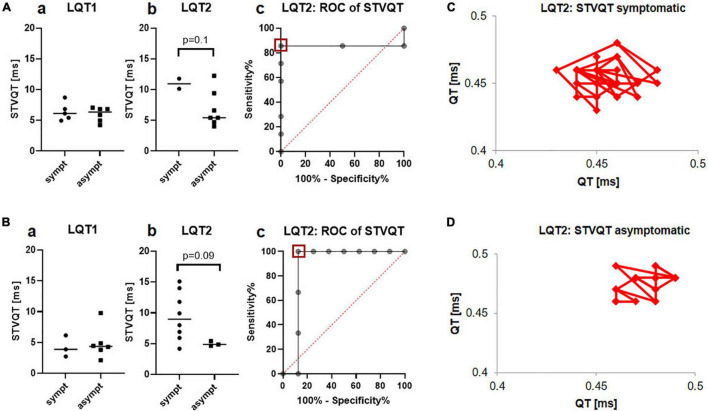
STVQT in the Holter ECG depending on the arrhythmic phenotype in LQT1 and LQT2. **(A)** Identification cohort from the University Hospital of Bern **a** No difference was observed between symptomatic (*n* = 5) and asymptomatic (*n* = 6) patients with LQT1 **b** STVQT tended to be higher in symptomatic (*n* = 2) compared to asymptomatic (*n* = 7) patients with LQT2 **c** ROC-analysis found a STVQT cut-off of less than 9.8ms (red square) identified symptomatic patients with LQT2 with a sensitivity of 85.7% and a specificity of 100%. **(B)** Validation cohort from the University Hospital Freiburg **a** STVQT did not differ between symptomatic (*n* = 3) and asymptomatic (*n* = 6) patients with LQT1 in the validation cohort **b** Validation cohort: STVQT also tended to be increased in symptomatic (*n* = 7) patients with LQT2 compared to asymptomatic (*n* = 3) controls. **c** ROC-analysis from STVQT in the validation cohort found cut-off of less than 5.7ms (red square) identified symptomatic patients with LQT2 with a sensitivity of 100% and a specificity of 87.5%. **(C)** Schematic representation of STVQT with Poincaré plots in a symptomatic LQT2 patient. QT intervals were plotted against the previous interval for 30 consecutive beats. Beat-to-beat variability is markedly enhanced compared to the asymptomatic patient depicted in **(D)**.

STVQT in the Holter-ECGs did not differ between symptomatic and asymptomatic patients with LQT1 (6.4ms ± 1.5ms vs. 6.0ms ± 1.2ms; Figure 5Aa), while it tended to be higher in the symptomatic compared to the asymptomatic LQT2 group (11.0ms ± 1.2ms vs. 6.8ms ± 3.0ms, *p* = 0.1; Figure 5Ab). Schematic Poincaré plots from one symptomatic and one asymptomatic patient are depicted in Figures 5C,D. ROC-analysis found a cut-off of less than 9.8ms identified asymptomatic patients with a sensitivity of 85.7% and a specificity of 100% (AUC 0.9, CI 0.6 to 1.0; Figure 5Ac). Due to the very small number of symptomatic patients with LQT2 that had received Holter-ECG monitoring, we decided to validate our findings in another cohort.

This validation cohort was recruited in the University Hospital of Freiburg and consisted of patients with genetically confirmed LQTS. The patients were categorized as symptomatic or asymptomatic according to the cohort from the University Hospital of Bern. The validation cohort consisted of 9 patients with LQT1 (3 symptomatic) and 11 patients with LQT2 (8 among them were symptomatic).

In the validation cohort from the University Hospital of Freiburg, STVQT also tended to be higher in the symptomatic patients with LQT2 compared with the asymptomatic group (9.5ms ± 3.9ms vs. 5.0ms ± 0.4ms, *p* = 0.09; Figure 5Bb). ROC-analysis found a STVQT cut-off in the validation cohort of less than 5.7ms to identify asymptomatic patients with LQT2 with a sensitivity of 100% and a specificity of 87.5% (AUC 0.9, CI 0.6 to 1.0; Figure 5Bc). Again, there was no difference between both groups in LQT1 (4.3ms ± 1.7ms vs. 4.9ms ± 2.6ms, Figure 5Ba).

## Discussion

In this study, we revealed a genotype-specific association between the classical ECG parameter QTc and of novel electrical markers for regional and temporal heterogeneity with the arrhythmic phenotype in LQTS. While the classical approach to assess QTc at rest could only determine the arrhythmogenic risk in LQT2 but not LQT1, QTc after exercise was a valuable marker to distinguish between symptomatic and asymptomatic patients with LQT1. Furthermore, parameters of regional electrical heterogeneity such as QT dispersion and delta T_peak/end_ were only associated with the clinical presentation in LQT1, but not in LQT2. Delta T_peak/end_ was the ideal parameter to distinguish between symptomatic and asymptomatic patients, especially after exercise. In contrast, parameters of temporal regional heterogeneity (STVQT) were linked to the arrhythmic phenotype only in LQT2. Our findings support the notion that risk stratification in LQTS should utilize electrical parameters in a genotype-specific manner.

### Corrected QT Interval

The global duration of the action potential is depicted by the QT interval in the surface ECG. In LQTS, QTc is a commonly accepted ECG marker not only for the diagnosis (13, 35) but also with prognostic implications (36). According to current guidelines, LQTS can be diagnosed clinically with either a QTc ≥ 480ms in repeated 12-lead ECGs or a Schwartz score > 3 which also includes QTc as a criterion (16, 35). In genotype-positive patients with LQTS, QTc prolongation was shown to be associated with arrhythmic risk (37, 38). However, there is evidence that QTc as a marker for arrhythmic risk has several limitations: QTc varies over time, depending on the autonomic tone and sex hormone levels (39, 40). Moreover, although QTc intervals are usually prolonged in LQTS-mutation carriers compared to healthy controls, there is substantial overlap in QT duration so that genotype-positive individuals can present with a normal QTc (41, 42). Even when they present a normal QTc interval, patients with LQTS still have a 10-fold increased risk for arrhythmic events compared to healthy controls (17). Therefore, current risk stratification based on QTc is often inaccurate.

In our study, QTc was similar among all LQTS genotypes, which is in line with previous findings from larger LQTS cohorts (38, 43). Remarkably, we observed that the QTc at rest did not differ between the symptomatic and asymptomatic patients with LQT1, while it was significantly associated with arrhythmic risk in the LQT2 group. An association of prolonged QTc with arrhythmic risk was described to be independent of the LQTS genotype (44). However, studies comparing QTc at rest from symptomatic and asymptomatic patients with LQTS described conflicting results. While Bekke et al. reported an association of QTc at rest with the clinical phenotype in LQT1 and LQT2 (38), Viitasalo et al. found no association of QTc at rest with the symptoms, neither in LQT1 nor LQT2 (45). In our cohort, QTc at rest was only associated with arrhythmic risk in LQT2. This discrepancy in different studies may also stem from the fact that QTc may show day-to-day variability in individual patients, for example, depending on their autonomic tone and/or hormonal state (39, 40).

In contrast to the QTc at rest, exercise-induced QTc prolongation could be observed in the symptomatic patients with LQT1, but not in the LQT2 cohort. This different response of LQT1 and LQT2 mutation carriers in response to sympathetic stimuli is due to the different underlying molecular mechanisms of LQT1 and LQT2. During adrenergic stimulation and exercise, the K^+^-channel Kv7.1 is phosphorylated and thereby activated leading to an increased I_*Ks*_ current density, thus rendering I_*Ks*_ a main repolarizing current during exercise/high sympathetic tone. In contrast, I_*Kr*_ is the main repolarizing current at rest (46, 47). There is a loss of function of I_*Ks*_ in LQT1, while I_*Ks*_ is not impaired in LQT2 (3), leading to a pathognomonic QTc prolongation during and after exercise in LQT1 but not LQT2. Our findings of a generally markedly prolonged QTc interval in symptomatic patients with LQT2 are in line with an impaired I_*Kr*_ function, while exercise-induced QTc prolongation associated with arrhythmic risk in LQT1 is due to the diminished I_*Ks*_ current (and therefore reduced capability for adrenergic I_*Ks*_ increase). In line with previous studies, exercise-induced QTc prolongation was only observed in LQT1, but not in LQT2 (43, 48–50). Exercise-induced QTc was therefore postulated as a useful tool to predict the genotype in patients suspected of LQTS (51). However, data on the superiority of post-exercise QTc on risk prediction in LQT1 were thus far missing, and to our knowledge, to date, no studies have focused on QTc differences between symptomatic and asymptomatic patients with LQT1 after exercise. ROC analyses revealed that a QTc cutoff of 488.5ms after 4 min of exercise had a valuable prognostic value with a sensitivity of 100% and a specificity of 75%, thus indicating the importance of exercise testing in risk prediction in LQT1.

### Parameters of Regional and Temporal Electrical Heterogeneity

There is emerging evidence that the electrophysiological substrate of arrhythmia formation is not only the extended duration of the action potential depicted by a prolonged QTc. Rather, an increased temporal and spatial dispersion of repolarization was described to be essential for the development of reentry-based arrhythmias (52, 53). We therefore evaluated ECG markers of regional and temporal electrical heterogeneity regarding their usefulness in genotype-specific risk prediction.

### Delta T_peak/end_

The T-wave in the ECG depicts the transmural repolarization of the ventricle. While the repolarization of epicardial cells is associated with the peak of the T wave, repolarization of the M cells (the cells between the endocardium and epicardium) coincidences with the end of the T-wave (54, 55). Thus, the interval between the peak and the end of the T-wave (T_peak/end_) is considered a measure of transmural dispersion of repolarization (54).

In congenital LQTS, a prolongation of T_peak/end_ was described with particular exercise-induced attenuation in LQT1 (21, 56, 57). Based on these findings and due to the fact that T_peak/end_ seems to be a reliable predictor for arrhythmic events in acquired LQTS (58), T_peak/end_ was hypothesized as a potential novel marker for risk prediction in congenital LQTS. However, large inter-patient variability in T_peak/end_ was postulated as a possible limitation of this parameter (59). In line with this, we did only observe an association of the arrhythmogenic phenotype with T_peak/end_ in LQT2 in the 12-lead ECG at rest. ROC analyses revealed that the sensitivity and specificity of this parameter to discriminate between symptomatic and asymptomatic patients is not sufficient to allow reliable discrimination between both groups.

A completely different situation emerged after a modification of the parameter T_peak/end_ with additional consideration of longitudinal repolarization gradients. Physiologically, there is an apico-basal dispersion in action potential duration (60). To depict this longitudinal gradient additionally to the transmural dispersion of repolarization, we calculated the delta T_peak/end_ between the more basally located lead V2 and the apically located V5 (T_peak/end_ V5 – T_peak/end_ V2). We observed that delta T_peak/end_ was associated with the arrhythmic phenotype in LQT1, with particularly good discrimination after exercise. All symptomatic patients with LQT1 had positive values and all asymptomatic LQT1 controls had negative values. Furthermore, delta T_peak/end_ was more negative in LQT2 compared to LQT1. Both could be a result of a different dispersion of I_*Ks*_ (that is defective in LQT1) and I_*Kr*_ (that is defective in LQT2) in the heart (60, 61). In rabbit hearts (which are similar to human hearts concerning the ventricular electrophysiology), I_*Ks*_ tail density is significantly reduced in the apex compared to the base, while I_*Kr*_ tail density varies in opposite apico-basal direction (61, 62). In line with this are findings from two studies that investigated not only the electrical but also the resulting mechanical alterations and described a significantly disturbed relaxation pattern in two cohorts of patients mainly with LQT1, where the relaxation at the base was delayed compared to the apex (63, 64).

Taken together, the novel parameter delta T_peak/end_ might indeed represent a valuable marker in genotype-specific risk stratification. With a sensitivity and specificity of 100% at a cut-off of −1ms for exercise-induced delta T_peak/end_ in our cohort, we clearly advocate for further evaluation of this novel parameter also in other patient cohorts. It seems to be a promising marker for genotype-specific risk stratification in LQT1, and it shows better reliability than the currently used QTc (which was clearly inferior regarding sensitivity and specificity according to ROC analysis).

### QT Dispersion

It was first hypothesized in 1988 by Cowan et al. that the QT inter-lead variability (QT dispersion) in a standard 12-lead ECG depicts the heterogeneity of ventricular repolarization and might therefore be a marker of electrical instability (65). In line with this theory, the QT dispersion was shown to be increased in patients with LQTS, and in several studies, a direct correlation between the degree of QT dispersion and arrhythmic events was observed (22, 66). Moreover, QT dispersion was significantly reduced in responders to beta-blocker therapy or sympathetic denervation, further supporting its causal connection with arrhythmic risk (22). Novel ECG techniques with higher spatial resolution, such as non-invasive mapping with ECG imaging, revealed that a steeper dispersion of repolarization could be detected in symptomatic patients with LQTS compared to an asymptomatic cohort (53, 67). However, none of these studies focused on genotype-specific differences in QT dispersion. In our study, QT dispersion was significantly more pronounced in symptomatic compared to asymptomatic patients with LQT1, with no differences in the LQT2 cohort.

### Short Time Variability of QT

Not only regional but also temporal instability of repolarization may contribute to arrhythmia formation. In the surface ECG, the temporal variation of repolarization can be quantified by calculating the short-term variability of the QT interval (20). STVQT as a marker for electrical instability is increased in patients prone to arrhythmias including acquired and congenital LQTS with further alterations in symptomatic individuals (20, 33). To our knowledge, our study is the first to describe genotype-specific associations of STVQT with arrhythmic risk in LQTS. We found that it might be a useful marker for arrhythmic risk in LQT2.

Our findings underline the need for a more individualized risk stratification in LQTS that should also account for genotype differences in the sensitivity and specificity of various electrical parameters for risk stratification.

### Limitations

The findings from our study are limited due to the small number of participants. The focus on a genotype-specific comparison of electrical parameters and their association with arrhythmic risk, however, is a strength of our analysis. To our knowledge, little was known about the usefulness of ECG markers of regional and temporal heterogeneity for genotype-specific risk stratification in LQTS prior to our study. We clearly advocate for conducting further studies in larger LQTS cohorts to validate our results.

## Data Availability Statement

The raw data supporting the conclusions of this article will be made available by the authors, without undue reservation.

## Ethics Statement

The studies involving human participants were reviewed and approved by the Cantonal Ethics Committee of Bern (Switzerland) (ID Nos: 2016-01602 and 2020-00316) and the Institutional Ethics Committee from the University Hospital of Freiburg (Germany) (ID 479/14). Written informed consent to participate in this study was provided by the participants or their legal guardian.

## Author Contributions

MR: study design, patient recruitment, acquisition, analysis and interpretation of data, and preparation of the manuscript. PK: acquisition, analysis and interpretation of data (validation cohort), and manuscript revision. JS, HS, AC, and BA: patient recruitment, acquisition of data, and manuscript revision (identification/validation cohort). DZ: acquisition and analysis of data (validation cohort) and manuscript revision. LN: acquisition of data and manuscript revision. CZ: genetic testing and manuscript revision. KEO: study design, patient recruitment, acquisition and interpretation of data, manuscript preparation and revision, and project supervision. All authors contributed to the article and approved the submitted version.

## Conflict of Interest

The authors declare that the research was conducted in the absence of any commercial or financial relationships that could be construed as a potential conflict of interest.

## Publisher’s Note

All claims expressed in this article are solely those of the authors and do not necessarily represent those of their affiliated organizations, or those of the publisher, the editors and the reviewers. Any product that may be evaluated in this article, or claim that may be made by its manufacturer, is not guaranteed or endorsed by the publisher.
