# Erratum: Genotype-specific ECG-based risk stratification approaches in patients with long-QT syndrome

**DOI:** 10.3389/fcvm.2023.1181494

**Published:** 2023-03-20

**Authors:** Frontiers Production Office

**Affiliations:** Frontiers Media SA, Lausanne, Switzerland

**Keywords:** long-QT syndrome, genetic arrhythmia disorders, risk stratification, QTc, electrocardiogram

An Erratum on Genotype-specific ECG-based risk stratification approaches in patients with long-QT syndrome By Rieder M, Kreifels P, Stuplich J, Ziupa D, Servatius H, Nicolai L, Castiglione A, Zweier C, Asatryan B and Odening KE (2022) Front. Cardiovasc. Med. 9:916036. doi: 10.3389/fcvm.2022.916036

An omission to the funding section of the original article was made in error. The following sentence has been added: “Open access funding was provided by the University of Bern”.

The original version of this article has been updated.

